# A Picture You Can Handle: Infants Treat Touch-Screen Images More Like Photographs than Objects

**DOI:** 10.3389/fpsyg.2016.01253

**Published:** 2016-08-22

**Authors:** Christine J. Ziemer, Makenna Snyder

**Affiliations:** Department of Psychology, Missouri Western State University, Saint JosephMO, USA

**Keywords:** infants, perception, touch-screens, perception and action, picture perception

## Abstract

Infants actively explore their world in order to determine the different ways in which they can interact with various objects. Although research on infant perception has focused on how infants understand the differences between 2- and 3-dimensional objects, today’s infants increasingly encounter 2D images with interactive qualities on smart-phone screens, tablets, and laptops. The purpose of this experiment was to examine the types of manual behaviors infants direct toward tablet images and to compare these actions to those evoked by 2D photographs or 3D when tactile feedback is controlled. Infants between the ages of 7–10 months sat on their parent’s lap in front of a table with a built-in well covered by a clear, plastic sheet while the three types of displays (photographs, objects, and screen images on a tablet) were presented for 30 s each. Infants saw three examples of each type of display presented in the built-in well so that tactile feedback information from the different displays was controlled. Coders noted the proportion of trials in which infants grasped, scratched, rubbed, or patted the display. Results indicate that infants direct significantly more grasps, scratches, and rubs toward 3D objects than 2D photographs. Infants also direct more grasps to objects compared to screen images. Our data suggests that infants are treating screen images more similarly to 2D photographs than 3D objects.

## Introduction

Is an object depicted on a touch-screen a picture, or an object? On one hand, it’s a flat, 2-dimensional (2D) surface; on the other, the object depicted on the screen may respond to your touch by moving, growing bigger or smaller, making noise, or performing some other function. Images displayed on a touch-screen exist in a new realm somewhere between 3-dimensional (3D) objects and static, 2D images. Items depicted on touch-screens do not afford the same type of manual exploration as a 3D object, yet they offer more interaction than a static, 2D photograph. The prevalence of this new technology provides an interesting question in the world of infant picture perception. What do infants who encounter this type of technology understand about the properties of touch-screen displays? Do the ways in which infants explore screen-projected images reflect an understanding of their interactive nature?

Infants actively explore their world through touch and hand manipulation in order to determine the ways in which they can interact with various 2D and 3D objects. They touch, pat, bang, scratch, rub, and grasp at objects, and in doing so, gain an understanding of the object’s properties and affordances for action. When manipulating images on a touch-screen phone or tablet, adults, and even young children display “screen-appropriate behaviors” (e.g., the “swipe,” the “flick,” and the “spread,” Cristia and Seidl, 2015), showing that they understand the different properties of interactive screen images as compared to static screen images or photographs. However, touch-screen technology is relatively new and little is known about how young infants perceive the different affordances of touch-screens as compared to photographs and objects.

Infants have been shown to be sensitive to visual cues to depth (e.g., relative size, linear perspective, shading, texture gradient, etc.) as early as 5 months (Gordon and Yonas, 1976; Kavšek et al., 2009, 2012). When viewing virtual objects designed to appear closer or further away from the infant, infants reach more frequently to the nearer appearing object (Gordon and Yonas, 1976). When monocularly viewing 2D displays designed to create the illusion, via pictorial depth cues, of one display being closer than the other, infants will reach preferentially toward the nearer looking display. However, when viewing the same displays with both eyes, young infants are not fooled by the visual illusion and do not show preferential reaching (see Kavšek et al., 2009 for a meta-analysis on infants’ sensitivity to pictorial depth cues via preferential-reaching studies).

As Goodale and Milner (1992) put forth, a dual-pathway visual system in the brain may explain the different reactions to stimuli seen as 3D (graspable) or 2D (non-graspable). Graspability dictates whether visual information is processed by the dorsal or ventral visual stream. If the object is perceived as being graspable, visual information will be processed dorsally (where binocular and motor responses are processed), if not, it will be processed ventrally (where pictorial information and perception judgments are processed). If infants as young as 5-month-old are sensitive to visual cues for depth, they should have a good sense of whether or not a display is 2D or 3D (i.e., not-graspable or graspable) based on depth cues if they are allowed to use both eyes to view the display. However, anecdotal reports of children “grasping” at 2D displays (images in a picture book or photographs) has prompted a line of research on this topic and suggests that there is still some manual exploration occurring as infants finalize their understanding of the different affordances of 2D and 3D objects.

A great deal of research in the field of infant picture perception has focused on understanding how infants perceive the differences between 2D and 3D objects and their ability to interact with and grasp these types of objects (e.g., DeLoache et al., 1998, 2003; Pierroutsakos and Troseth, 2003; Yonas et al., 2005; Ziemer et al., 2012). DeLoache et al. (1998, 2003) use the phrase “pictorial competence” to describe when infants understand that a picture is both an object in and of itself as well as a representation of what it depicts. When infants achieve this understanding of the dual nature of pictures and photographs they can begin to focus on the abstract, representational nature of photographs instead of the concrete aspects of the photograph itself (DeLoache et al., 1998). Therefore, younger children exhibit more manual exploration of photographs through rubbing, patting, and sometimes appearing to grasp at the images depicted; while older children respond with less manual exploration overall and exhibit more picture-appropriate behaviors such as pointing to the image (DeLoache et al., 1998; Pierroutsakos and Troseth, 2003).

Upon further investigation, Yonas et al. (2005) found that, when comparing the way 9-month-old infants reached toward various 2D depictions and objects, the shape of the infant’s hand as well as the angle of the reach changed when infants were reaching toward a 2D depiction versus a 3D object. Infants reached with their hands higher when approaching an object than when approaching a photograph of an object. The angle and height of infants’ reaching did not change when reaching for a photograph of an object compared to a non-pictorial (abstract) 2D display. This change in the hand approach for objects versus 2D displays indicates that infants may not be trying to “grasp,” or pick up, the objects depicted in 2D images as DeLoache et al. (2003) suggest.

More recently, Ziemer et al. (2012) compared the manual behaviors that 9-month-old infants exhibited toward 3D objects and highly realistic 2D photographs when tactile feedback was controlled. Infants were presented with photographs and objects presented one-at-a-time under a Plexi-Glas^®^ surface which covered a built-in well in the surface of a table. Coders noted the presence or absence of four types of actions—grasps, pats, rubs, and scratches—that infants directed toward the photograph and objects under glass. Ziemer et al. (2012) found that rubbing was the most frequent action followed by patting. For both of these frequent behaviors, there was no significant difference between the amount of rubs and pats directed toward 3D objects as compared to 2D photographs. However, when it came to the behaviors that might be considered more 3D-appropriate (grasps and scratches), Ziemer et al. (2012) found that 9-month-old infants directed significantly more of these behaviors toward objects than toward photographs. They concluded that by 9 months of age, infants are able to recognize and respond appropriately to the 2D photographs and 3D objects.

Today’s infants are born into a world in which touch-screen technology is more prevalent than ever before. Parents and older siblings may have touch-screen phones and/or tablets that they use not only for voice calls, but video calls, taking photographs, and videos, checking weather, reading, and sending e-mails, playing games, and listening to music among many other functions. Infants are encountering interactive touch-screens with greater frequency and at earlier and earlier ages (Cristia and Seidl, 2015). Arguably, touch-screen displays are outside the scope of previous research on infants’ understanding of the differences between 2D and 3D pictures and objects. Touch-screen images are 2D pictures projected on a flat surface yet they are able to be manipulated by touch. Therefore they are unlike static photographs and drawings and different from passive screen-images infants may encounter on television and movies. Touch-screen images break the rule that 2D depictions do not afford manual manipulation because they respond to specific forms of touch and encourage manual exploration.

In 2003, before touch-screen technology was as prevalent as it is today, Pierroutsakos and Troseth (2003) conducted a study examining the actions that infants direct toward stationary and moving videos presented on screens. They found that older infants (15 and 19 months old) exhibited less manual investigation and more pointing and vocalizing behaviors when exploring a screen image compared to 9-month-old infants who commonly grasped at, hit, and pat an image depicted on a screen. Pierroutsakos and Troseth (2003) also noted that the manual investigation behaviors that infants *do* display toward screen images may not be caused by infants confusing 2D images for 3D objects as the infants expressed little surprise or frustration at their inability to pick up an image. Rather, infants may be merely exploring the ways in which an image may be manipulated and learning about the concept of pictorial representation (Pierroutsakos and Troseth, 2003). A tendency for infants to manually explore flat surfaces may be especially useful as infants learn to use touch-screens that *can* be manipulated and respond to tactile interaction.

To date, there has been little descriptive or experimental research focusing specifically on infants’ understanding of touch-screens as this technology as a common household item is fairly new. Research that has examined infants’ exploration of screens has focused on passive screens which sit upright, facing the infant (like a television screen, e.g., Pierroutsakos and Troseth, 2003) instead of flat on a table as tablets and other touch-screens are usually used. Understanding how infants interact with touch-screen images is of growing importance. Both passive and interactive screen products such as movies, books, and games are being marketed for infants at a growing rate (Pierroutsakos and Troseth, 2003; Cristia and Seidl, 2015). Research into how infants understand this new kind of stimuli lags behind the creation of these programs. Currently, the American Academy of Pediatrics (AAP) recommends no screen time for children under the age of two; however, these guidelines came out before the release and popular usage of the iPad and tablets (Christakis, 2014). In order to understand the benefits or drawbacks of touch-screen products for young infants, we must first understand how infants perceive these screen images and how they fit into infants’ schemas of 2D and 3D objects.

The following experiment examines how infants raised in today’s culture perceive and interact with images presented on a tablet screen. The aim of this study was to determine if infants treat screen-displayed images more like passive 2D photographs or like interactive 3D objects. We observed the manual behaviors infants directed toward screen images on a tablet and compared them to the behaviors evoked by 2D photographs and 3D objects. Our methods replicate and extend those used by Ziemer et al. (2012) by controlling for tactile feedback with all three types of stimuli.

## Materials and Methods

### Participants

Twenty-one infants (12 females) between the ages of 7-months and 10-months participated in this experiment. One infant was not included in the analyses because he was born over two months premature. The mean age of infants included in the analyses was 8 months 26 days. This age range was chosen in order to compare with previous infant picture perception research (e.g., DeLoache et al., 1998; Pierroutsakos and Troseth, 2003; Yonas et al., 2005; Ziemer et al., 2012) and because infants at this age have good depth perception and will reach for and explore objects (Gordon and Yonas, 1976; Yonas and Hartman, 1993; Kavšek et al., 2009). Infants were recruited for participation through an email sent to faculty at a Midwestern university, online Facebook groups for parents, and postings at local libraries and daycares.

### Materials

Parents completed a questionnaire with nine items to assess screen usage and exposure. Three items examined how often the infant played games, watched movies, and played with a powered off device (e.g., Please indicate how often your child uses the following devices to play games or use apps). With these questions, a list of devices was given (TV/Video Games, Computer/Laptop, Tablet/iPad, and Cell Phone). For each device, activity was measured on a five-point scale from “never” to “several times a day.” One item asked parents how often their infant operated devices without adult assistance. Two items explored infant exposure to screens without direct use through the amount of time primary caregivers spend on devices and number of screens (e.g., smart phones, televisions, computers, and tablets) in the home.

Objects, photographs, and screen images were presented to the infants during the experiment. Objects consisted of nine small infant toys with bright colors designed to attract an infant’s attention. The photographs depicted the same nine toys printed on glossy white paper and affixed to foam board. These same nine images were also loaded onto a tablet device (Amazon Fire, 7-inch display) to create the screen images. Infants were shown three of each format– a total of nine trials for each infant. Objects, photographs, and screen images were all roughly the same size (approximately 4 inch × 3 inch).

The objects, photographs, and screen-images were presented in a table with a square, built-in well (8.5 inch × 8.5 inch) which was covered by a clear plastic (Plexi-Glass^®^) sheet. A white cushion in the well beneath the objects, photographs, and tablet allowed the displays to sit right up against the Plexi-glas^®^. The Plexi-glas^®^ sheet was attached to the table with a hinge on the infant’s side allowing the experimenter to raise and lower the sheet in order to change the display between trials. While the display was changed, a colorful piece of tag-board covered the Plexi-Glas^®^ keeping the infant from observing the experimenter.

Thirty-second trials were timed by the experimenter using a hand-held stopwatch. Sessions were recorded using two Logitech web-cams (Tessar 2.0/3.7, 2MP autofocus) and the Panopto recording program (Windows computer compatible). Recordings were saved to a private Panopto folder only accessible to the experimenters.

### Procedure

After signing the consent document and filling out the questionnaire on screen-use, infants and parents were brought into the lab. Infants sat on their parent’s lap in front of a small table. The experimenter sat directly across the table from them. Parents were instructed to hold their child on their lap with their hands around their child’s waist and not to interfere with their child’s arms or touch the table themselves at any point. A short warm-up toy interaction between the experimenter and the child was used to make sure infants were able to reach the table where the displays would be presented. Parents were not informed about the purpose of the study, but were reminded that there was no “right” or “wrong” behavior their child should be exhibiting. It was also explained that their child was not participating in an assessment; rather their responses were merely being observed in order to gain a better understanding of infant perception.

Items were presented one-at-a-time for 30 s each. Each infant saw three photographs, three objects, and three tablet images in a randomized order. The presentation order as well as format for each toy presentation was randomized for each participant. Infants saw one version of each of the nine toys (i.e., they did not see both the photograph and object/screen image of the same toy). Each object was placed in the well on a cushion, so that it was directly beneath the Plexi-Glas^®^ sheet. Photographs were attached to a piece of foam-board that fit the edges of the well, so they were pressed up to the plastic sheet as well. The tablet was also displayed on the cushion along with a piece of white poster board cut to frame the size of the image on the screen and to block the edge of the tablet from view.

Infants were allowed to explore each item for 30 s. If they seemed to have not noticed the item (e.g., had not looked at the item), the experimenter tapped on the table and verbally directed the infant’s attention to the item. If an infant became fussy during the session, he or she would be allowed to take a break and then try to resume the session. Sessions were recorded from two different angles to allow different views for coding. At the end of the session, infants were given a toy to thank them for their participation.

### Coding

Infants’ manual behaviors toward the photographs, objects, and screen images were coded from the video recordings. Only manual behaviors that came in contact with the well area around the stimuli were counted. Coders recorded the presence or absence of pats, rubs, scratches, and grasps for each of the nine test trials. Pats were hand movements that came in contact briefly with the surface of the table (above the well area), either lightly touching or slapping. Rubs were hand movements that swept across the table coming in contact with the well area during some part of the movement. Scratches were hand movements in which one or more of the infant’s fingers (usually the index finger) flexed and extended while in contact with the surface. Grasps were hand movements in which the infant’s four fingers and thumb flexed closed into a fist while in contact with the table surface (see Butterworth et al., 1997; Ziemer et al., 2012). Coding for each trial began when the infant first looked at the photograph, object, or screen image and ended when the stimuli was covered up between trials. Inter-coder reliability (*N* = 4 infants) based on exact percent agreement (i.e., whether each action was present of absent on each trial) was 94.44%.

Proportion scores were computed by taking the number of trials of each type (photograph, object, and screen image) in which a given action occurred divided by the number of trials an infant completed for each trial type (see Yonas et al., 2005; Ziemer et al., 2012). The use of proportion allows even occasional behaviors to be represented in the data and is less subjective than counting the number of occurrences for each individual behavior.

## Results

Parents of the infants in this study reported having an average of 5.65 screens in their home (*SD* = 2.01, range 3–11). This included televisions, computers, smart phones, and tablets. The most common ways that parents reported their infants interacted with screen devices at home was by playing with powered-off phones and watching movies on a television. None of the parents in this study reported that their infants had ever used games or applications on a tablet, although a few parents reported that their children played games or applications on a phone, computer, or television. A few parents also reported that their infants occasionally watched movies on a computer or tablet and also interacted with these screens while the device was powered off. None of the parents included in the analysis reported that their infant ever used a screen device without supervision.

**FIGURE 1:**
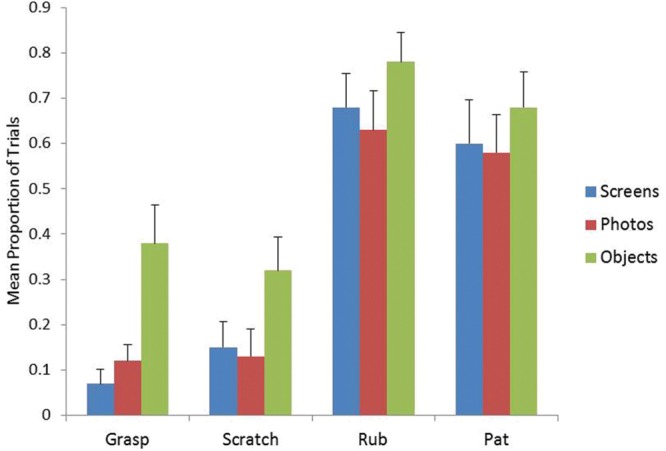
**Mean proportion of grasps, scratches, rubs, and pats directed to screens, photographs, and objects**.

**Figure 1** shows the mean proportion of trials in which infants directed grasps, scratches, rubs, and pats toward photographs, objects, and screen images. Overall, the most frequent action was rubbing, followed by patting. Grasping and scratching were relatively infrequent. The means proportion of trials infants grasped at the display was 0.07 (*SD* = 0.137) for screen images, 95% CI [0.01, 0.13], 0.12 (*SD* = 0.163) for photographs, 95% CI [0.05, 0.19], and 0.38 (*SD* = 0.379) for objects, 95% CI [0.21, 0.55]. The mean proportion of trials infants scratched at the display was 0.15 (*SD* = 0.253) for screen images, 95% CI [0.04, 0.26], 0.13 (*SD* = 0.274) for photographs, 95% CI [0.01, 0.25], and 0.32 (*SD* = 0.333) for objects, 95% CI [0.17, 0.47]. The mean proportion of trials infants rubbed the display was 0.68 (*SD* = 0.333) for screen images, 95% CI [0.53, 0.83], 0.63 (*SD* = 0.388) for photographs, 95% CI [0.46, 0.80], and 0.78 (*SD* = 0.292) for objects, 95% CI [0.65, 0.91]. The mean proportion of trials infant patted the display was 0.60 (*SD* = 0.427) for screen images, 95% CI [0.42, 0.79], 0.58 (*SD* = 0.373) for photographs, 95% CI [0.42, 0.74], and 0.68 (*SD* = 0.350) for objects, 95% CI [0.53, 0.83].

In order to determine whether 7–10-month-old infants performed different manual behaviors toward different types of depictions we first entered the proportion of grasps, pats, rubs, and scratches into an Display Type (3) × Action (4) repeated measures analysis of variance (ANOVA). The analysis yielded a significant effect of display type, *F*(2,38) = 17.82, *p* < 0.0001, and action, *F*(3,57) = 23.71, *p* < 0.0001. There was no significant Display × Action interaction *F*(6,114) = 1.32, *p* = 0.253, *ns*. Follow-up comparisons using the Bonferroni adjustment for multiple comparisons revealed that infants were rubbing significantly more than they were grasping (*p* < 0.001) and scratching (*p* < 0.001). Infants were also patting significantly more than they were grasping (*p* < 0.001) and scratching (*p* < 0.01). Infants directed significantly more behaviors toward objects than they did toward photographs (*p* < 0.001) or screen images (*p* < 0.001).

Protected paired-samples *t*-tests further revealed that infants directed a significantly higher proportion of grasps toward objects than they did toward photographs, *t*(19) = -2.89, *p* = 0.009, 95% CI [-0.46, -0.07], or screen images, *t*(19) = 3.71, *p* = 0.001, 95% CI [0.14, 0.50]. Infants also directed more scratches toward objects compared to photographs, *t*(19) = -3.24, *p* = 0.004, 95% CI [0.30, 0.06] and more rubs toward objects compared to photographs, *t*(19) = -2.93, *p* = 0.009, 95% CI [-0.26, -0.04]. No other differences were significant.

## Discussion

This experiment was designed to compare the manual behaviors infants displayed toward touch-screen images to the behaviors they direct toward objects and photographs. The infants in this study did not discriminate between the screen-images and photographs, showing similar types and amounts of behaviors toward screens as they did toward 2D photographs. Infants clearly showed a difference in the way in which they interacted with 3D objects as compared to photographs by directing more grasps, scratches, and rubs to objects than photographs. These results replicate the work by Ziemer et al. (2012) showing that, when tactile information is controlled, infants display different types of manual behaviors toward 2D and 3D displays. With the addition of a screen-image display, we were able to compare how, if at all, infants modify their behavior when a 2D image is presented on a screen. Our results indicate that, by 7–10 months, infants appear to understand that a screen-image is 2D, like a photograph. However, they do not appear to understand the interactive nature of touch-screens at this point in development.

One limitation of this sample was the fact that the infants tested had had little exposure to touch-screens. Although all of the families included in this study reported having several screens in their home, infants were not yet using these screens for interactive purposes. Movies and powered-off devices were the most frequent way infants were interacting with screens. Although it is possible (and quite likely) that the infants in this study have encountered social modeling by adults and older children interacting with touch-screen devices, the fact that they themselves had had little experience with the interactive nature of touch-screens may explain why the infants in this sample did not try to interact with the screen images more than static photographs.

In the future, it would be beneficial to our understanding of infant screen perception to recruit a more varied sample which better represents the population. The parents in our sample reported much less infant screen exposure than what has been reported in previous survey data (e.g., Kabali et al., 2015). This difference may have been caused by selection bias (e.g., the parents who had the time and inclination to bring their children to the university for a psychology study may be different from the population at large), or by bias in parents’ reporting (e.g., under-reporting or under-estimating the amount of screen exposure their child has in order to be seen more positively). On the other hand, if, with a more varied sample, we still find little use of screens within this age group, it calls into question the findings from previous literature that infant screen use is as widespread as has been claimed (e.g., Kabali et al., 2015 finding that 43.5% of children under 1 year use a mobile device daily). Making the experimental set-up portable and taking it to places where more varied samples of children may gather (e.g., daycares, libraries, and preschools), may increase the sample variability with regard to screen exposure. This is one of the directions we are pursuing for future research in this area.

By 9 months, most infants have fairly good control over their arms and hands, but are still mastering fine motor movements of the fingers. This is the age at which infants begin developing the “Pincer grasp” which utilizes the thumb and index finger to pick up a small object such as a Cheerio (Gesell, 1952). It may be the case that the types of behaviors we exhibit toward touch-screens are too fine or complex for young infants to display even if they wanted to. Cristia and Seidl (2015) identified screen-specific behaviors such as “swipe,” “flick,” “tap,” “press and drag,” “pinch,” and “spread,” and asked parents in their study to report the frequency with which their children used these gestures while interacting with screens. Although some of these screen-gestures were very common (68% reported children doing a “flick,” 71% reported the “tap”), the researchers noted that these behaviors, especially the more complex “pinch” and “spread,” increased with children’s age. Older children, with better dexterity and perhaps more screen experience, exhibited these behaviors with greater frequency than younger children (Cristia and Seidl, 2015).

In response to the concern that younger children may not have the dexterity to perform specific screen-appropriate manual behaviors toward an image depicted on a touch-screen, we are currently running a second group of infants between the ages of 15–18 months in the same experiment. A survey of parents by Kabali et al. (2015) found that 28.2% of 2-year-olds did not need any help navigating a mobile media device. It seems likely that between the ages of 1 and 2, as children’s dexterity and cognitive ability increases, they also learn a great deal about the different ways that touch-screens afford interacting as opposed to pictures or photographs. Consequently, infants in this older age group may show more or different types of manual investigation when exploring an image depicted on a screen. Alternatively, as Pierroutsakos and Troseth (2003) found, older infants may display *less* manual investigation of a screen surface if they have learned that these types of images do not afford manipulation.

Although the parents whose children participated in this study reported that they were limiting their infants’ exposure to screens before two years, many parents are either unaware or unable to stick to so strict a policy as put forth by the AAP. A survey of 350 children aged 6 months to 4 years by Kabali et al. (2015) found that 43.5% of children less than a year old and 76.6% of 2-year-old children used a mobile device daily to play games, watch videos, or use apps. Use of mobile devices by infants was not associated with child gender, ethnicity, or parent education. Parents in this study reported using the mobile devices to keep children entertained in order to do chores (70%), run errands (58%), calm their child down in a public setting (65%), or help their child fall asleep (28%; Kabali et al., 2015).

To be fair, it is nearly impossible to keep a child completely screen-free in today’s society. Screens are everywhere—in the hands of adults making calls and taking photographs of their infant, on the laptop where relatives have FaceTime or Skype conversations with infants, televisions are becoming common fixtures in restaurants and waiting rooms (even some gas station pumps now show advertisements on built-in screens while the pump is running). With so many screens around, it is important to understand what effects different types of media may have on the development of young children and infants.

For example, the video deficit effect, (e.g., Zack et al., 2009), indicates that infants learn less from television and 2D images than from face-to-face interactions, suggesting an inability for transfer what is learned on screens into the real world. Further research is necessary to explain if a similar deficit in learning occurs with *interactive* 2D sources, such as touch-screen tablets. Concerns regarding the usage of iPads and tablets extend beyond the influx in tablet marketing for infants. The use of screens for purposes other than entertainment, such as regulating your child’ mood, could have important implications for social and emotional development (Radesky et al., 2015). Impairment of the executive brain functions, which may be connected to ADHD, is implicated in screen overuse (Christakis, 2014). Researchers also fear that the use of iPads could inappropriately displace other enriching activities that provide active visual, language, and motor development (Radesky et al., 2015).

The results of this experiment indicate that, by 7–10 months, infants show little difference in their manual explorations of screen-projected images and 2D photographs. Although they occasionally may grasp or scratch at a screen image or photograph these behaviors are relatively rare and occur with much more frequency toward 3D objects. Our results suggests that infants are able to correctly perceive the flat surface of the screen and are not attempting to try to pick up the depicted object, treating it in the same way they treat a 2D photograph. To return to the idea of the dorsal/ventral visual pathway system (Goodale and Milner, 1992), it would appear that infants in our study were judging the screen images to be non-graspable (thus processed by the ventral stream as photographs are), rather than graspable (processed by the dorsal, action stream as objects may be). However, we still have much to learn about the ways in which young infants understand screen images and further experimental research examining different ages, different levels of screen experience, and different types of interaction with screens as well as long-term research into the effects of screen exposure during early years is needed to best advise parents of how to navigate this new world of touch-screens.

## Author Contributions

Both CZ and MS were involved in the design of this experiment, data collection, coding, data analysis, and writing of this document.

## Conflict of Interest Statement

The authors declare that the research was conducted in the absence of any commercial or financial relationships that could be construed as a potential conflict of interest.
